# The R2R3-MYB Factor FhMYB5 From *Freesia hybrida* Contributes to the Regulation of Anthocyanin and Proanthocyanidin Biosynthesis

**DOI:** 10.3389/fpls.2018.01935

**Published:** 2019-01-07

**Authors:** Yueqing Li, Xiaotong Shan, Liudi Zhou, Ruifang Gao, Song Yang, Shucai Wang, Li Wang, Xiang Gao

**Affiliations:** ^1^Key Laboratory of Molecular Epigenetics of MOE and Institute of Genetics and Cytology Northeast, Normal University, Changchun, China; ^2^National Demonstration Center for Experimental Biology Education, Northeast Normal University, Changchun, China

**Keywords:** general flavonoid pathway, MYB transcription factor, tobacco, transient transfection assay, protoplast

## Abstract

The flavonoids are important and nourishing compounds for plants and human. The transcription regulation of anthocyanin and proanthocyanidin (PA) biosynthesis was extensively studied in dicot compared with monocot plants. In this study, we characterized the functionality of an R2R3-MYB gene *FhMYB5* from the monocotyledonous flowering plant of Iridaceae, *Freesia hybrida.* Multiple sequence alignment and phylogenetic analysis implied that FhMYB5 was clustered into grapevine VvMYB5b subclade. Correlation analysis indicated that the spatio-temporal expression patterns of *FhMYB5* coincided well with anthocyanin and PA accumulations in *Freesia per se*. Furthermore, transient transfection assays in *Freesia* protoplasts revealed that the late flavonoid biosynthetic genes (e.g., *DFR* and *LDOX*) were slightly up-regulated by *FhMYB5* alone, whereas both early and late biosynthetic genes were significantly activated when *FhMYB5* were co-infected with either of the two IIIf clade bHLH genes, *FhTT8L* and *FhGL3L*. Moreover, these results were further confirmed by co-transfection of *FhMYB5* with either of the bHLH genes aforementioned into protoplasts expressing *GUS* reporter gene driven by *Freesia* promoters. In addition, the overexpression of *FhMYB5* in tobacco and *Arabidopsis* could also significantly up-regulate the expression of genes participating in the general flavonoid pathway. In conclusion, *FhMYB5* was proved to function in the general flavonoid pathway in *Freesia*. The results implied a function conservation of flavonoid biosynthesis related MYB regulators in angiosperm plants.

## Introduction

Anthocyanins and proanthocyanidins (PAs) are two major classes of flavonoids in plants. Anthocyanins, responsible for the wide range of colors including red, blue, violet and purple in plants, are implicated in protecting plants against damaging coldness and UV irradiation and facilitating pollination and seed distribution (Shao et al., 2018). PAs, also known as condensed tannins (CTs), are widely characterized by their abilities of anti-microbial pathogens, insect pests, and herbivores (Shao et al., 2018). Evolutionarily, the two kinds of specialized metabolites emerged at different times. The anthocyanins are widely spread in angiosperms while the PAs could be traced back in ferns (Koes et al., 1994). There is no doubt that the evolutionary emergence of anthocyanins and PAs have aided the plants in environment adaptation. Besides, there are growing evidences showing that adequate dietary intake of foods rich in powerful antioxidants, e.g., anthocyanins and PAs, can notably reduce the risk of sufferings from cardiovascular and immune diseases (Bagchi et al., 2003; Jang et al., 2010). Thus, the health-promoting athocyanins and PAs have hitherto attracted the attentions of both scientists and breeders (Albert, 2015; An et al., 2015, 2017; Huang et al., 2015; Gao et al., 2016).

The biosynthesis of anthocyanins and PAs has been widely investigated in plants over the past few decades. Two typical classes of genes, structural genes encoding enzymes accounting for anthocyanin and PA accumulations directly and regulatory genes encoding transcription factors regulating the structural genes at the transcriptional level, have been characterized in numerous plants (Hichri et al., 2011a; Jaakola, 2013; Liu et al., 2015; Xu et al., 2015; Zhao and Tao, 2015). More precisely, at least two groups of structural genes, namely the early and late biosynthetic genes (*EBGs* and *LBGs*) have been characterized in flavonoid biosynthesis (Lepiniec et al., 2006; Petroni and Tonelli, 2011). The *EBGs* contribute to the biosynthesis of dihydroflavonols which are the common precursors of several kinds of flavonoids, whereas the *LBGs* are more specific to anthocyanin and PA biosynthesis. Furthermore, these structural genes are finely regulated by transcription factors encoded by regulatory genes (Broun, 2005; Xu et al., 2015). Recent studies have pinpointed that a series of regulatory genes encoding R2R3-MYB regulators, basic helix–loop–helix (bHLH) transcription factors, WD40 proteins and other regulators are involved in the regulation of anthocyanin and PA biosynthesis (Jaakola, 2013; Xu et al., 2015). In the model plant *Arabidopsis*, partially redundant R2R3-MYB regulators AtMYB11, AtMYB12, and AtMYB111 can activate the *EBGs*, while a ternary complex composed of R2R3-MYB regulator AtPAP1, bHLH factor AtTT8 or AtGL3, and WD40 protein AtTTG1 mainly regulate the expressions of the *LBGs* in anthocyanin biosynthetic pathway (Baudry et al., 2004; Stracke et al., 2007; Gonzalez et al., 2008; Zhou et al., 2012; Jaakola, 2013; Xu et al., 2015). Meanwhile, another R2R3-MYB factor AtTT2 mainly regulates the PA-specific *AtBAN* by forming another complex with AtTT8 and AtTTG1 (Hichri et al., 2011a; Zhao and Tao, 2015). To date, the evolutionarily conserved regulatory protein complex partners have been widely characterized in various species such as petunia (*Petunia hybrida*), pear (*Pyrus bretschneideri* Rehd.), grape (*Vitis vinifera*), apple (*Malus domestica*), strawberry (*Fragaria x ananassa*) and other angiosperms, mainly in core eudicot plants (Petroni and Tonelli, 2011; Jaakola, 2013; Schaart et al., 2013; An et al., 2015; Pillet et al., 2015; Zhai et al., 2016). In the MBW complex, the widely accepted mainstream opinion is that MYB protein determines the target genes to be specially activated. Generally, the function of a specific MYB protein is usually limited to a specialized pathway, such as AtPAP1 for anthocyanins and AtTT2 for PAs (Liu et al., 2015). In contrast, some general MYB regulators have also been found in addition to these specialized MYB regulators, although the numbers are limited. VvMYB5a and VvMYB5b isolated from grapevine, and EsMYB9 characterized from *Epimedium sagittatum* are examples of this kind of MYB regulators controlling more than one branches of the flavonoid pathway (Deluc et al., 2006, 2008; Hichri et al., 2011b; Huang et al., 2012, 2017). Though, numerous MYB members have been well studied in several monocotyledon plants, such as *Zea mays, Oryza sativa, Lilium* spp. and *Phalaenopsis* spp., they were proved to regulate the specialized flavonoid biosynthesis (Grotewold et al., 1994; Maeda et al., 2014; Yamagishi et al., 2014; Hsu et al., 2015). Few general MYB regulators have been isolated from monocot plants, which is a constraint to clarify their functional conservation or difference in angiosperms.

*Freesia hybrida*, a monocotyledonous flowering plant of Iridaceae, is distinguished for its abundant flower colors and fragrances. Recent studies revealed that there were five kinds of anthocyanin aglycons, namely delphinidin, petunidin, malvinidin, peonidin, and cyanidin, and two kinds of flavonols, namely kaempferol and quercetin, as well as PAs in *F. hybrida* “Red River^®^” (Sun et al., 2015, 2016; Li et al., 2016). To date, six structural genes and two IIIf bHLH regulatory genes have been functionally characterized (Sui et al., 2011; Sun et al., 2015, 2016, 2017; Li et al., 2016, 2017; Ju et al., 2018). In this study, the first MYB regulatory gene *FhMYB5* was isolated and performed as candidate gene involved in flavonoid biosynthesis in the *Freesia* flowers. Spatiotemporal expression analysis implied a potential correlation of *FhMYB5* with both anthocyanin and PA accumulations in *Freesia per se*. Transient expression assays indicated that FhMYB5 was an activator and participated in both anthocyanin and PA biosynthesis by interacting with bHLH co-factors. This was further validated by introducing *FhMYB5* into tobacco as well as *Arabidopsis*, representatives of Asterids and Rosids, respectively. This study not only clarified the molecular mechanisms of flavonoid biosynthesis in *Freesia*, but also preliminarily indicated the evolutionary conservation of flavonoid biosynthesis related MYB proteins between monocots and dicots.

## Materials and Methods

### Plant Materials and Growth Conditions

One cultivar of *F. hybrida*, named Red River^®^ was used in this study. Bulbs were grown in sandy soil in a greenhouse at 15°C under 14 h/10 h (light/dark) photoperiod. The soil was watered aperiodically to keep moist before anthesis (approximately one time a week). Young inflorescence segments were surface sterilized and employed as explants to induce calli following the highly efficient systems established in wild species and modern cultivars (Gao et al., 2010; Uwagaki et al., 2015), and the calli were subjected to protoplast isolation and transient transfection analysis. Moreover, flowers at different developmental stages and tissues stripped from the fully blooming flowers were randomly sampled and immediately frozen in liquid nitrogen before storage at -80°C, which were subsequently used for gene cloning, spatio-temporal expression analysis as well as promoter isolation.

*Nicotiana tabacum* (*cv. K326*) and *Arabidopsis thaliana* (*Columbia-0*) were grown in a greenhouse at 22°C under a 16 h light/8 h dark regime. The tobacco flowers fully blossomed at the first day were harvested and divided into five tissues, i.e., toruses, calyxes, petals, stamens and pistils. And the fully expanded mature leaves, the fourth or fifth leaf from the apical meristem, were sampled. The samples were immediately frozen in liquid nitrogen and stored at -80°C before further analysis. Moreover, 4-week-old *Arabidopsis* leaves were chosen for flavonoid accumulation analysis, gene expression analysis and protoplast isolation.

### DNA and RNA Extraction and cDNA Synthesis

Plant samples grounded into powder in liquid nitrogen were processed by NuClean Plant Genomic DNA Kit (CWBIO, Beijing, China) for DNA extraction referring to the manufacturer’s instruction. Total RNA was isolated from various tissues using the OminiPlant RNA Kit (CWBIO, Beijing, China) followed by DNase I digestion to remove DNA contamination according to the manufacturer’s instruction. The purity and concentration of RNA was then assessed by NanoDrop 1000 spectrophotometry (Thermo Scientific, United States). 1 μg of total RNA was reversely transcribed into cDNA using UEIris II RT-PCR System for First-Strand cDNA Synthesis Kit (US Everbright^®^ Inc., Suzhou, China).

### Gene and Promoter Cloning

*Freesia* transcriptomic database was constructed by the high throughput sequencing of RNA samples extracted from flower tissues including toruses, calyxes, petals, stamens, and pistils and flowers at different developmental stages. *In situ* TBLASTN screen was conducted using *Vitis* VvMYB5a (GenBank accession number: AAS68190) as probe bait. The candidate gene was amplified using specific primers (Supplementary Table S1) designed according to the predicted sequence, and cloned into pGEM-Teasy vector (Promega, Madison, WI, United States) for sequencing confirmation. The verified gene sequence was subsequently subjected to manual BLASTX search of NCBI database for further analysis (Supplementary Table S2).

The promoters of *Freesia* anthocyanin biosynthetic genes were cloned from genomic DNA isolated with Genome Walking Kit (Takara, Dalian, China). At least three specific reverse primers (SP primers) for each gene were designed for TAIL-PCR with arbitrary degenerate primers (AP primers supplied in the kit) to isolate upstream sequences (promoters) according to the manufacturer’s instructions. The PCR products were sub-cloned into pGEM-Teasy vector and sequenced. Finally, 1,000–1,500-bp upstreams of the ATG transcriptional start site were tentatively employed as promoter sequences. The primers were presented in Supplementary Table S1.

### Plant Transformation

About 5- to 6-week-old tobacco was transformed and regenerated according to Sparkes et al. (2006). The binary vectors were introduced into *Agrobacterium tumefaciens* strain GV3101 and injected into tobacco leaves with syringes. About 5 days later, the infiltrated leaves were sterilized and cut into pieces. The leaf squares were then placed on the shooting medium with 50 μg mL^-1^ kanamycin until shoots appeared. Three to four weeks later, the shoots were transferred onto the rooting medium, and roots would appear within 7–10 days. The rooted plantlets were deemed as T0 generations and then transferred into pots and grown in the greenhouse. Fully expanded mature leaves and blooming flowers of T0 tobacco plants were collected for further analysis.

About 5- to 6-week-old *Arabidopsis* with a few mature flowers was transformed with GV3101 containing *FhMYB5* by the floral dip method (Clough and Bent, 1998). Selection of the T1 transformants was carried out on 1/2 MS supplemented with 50 μg mL^-1^ kanamycin, and the survivals were transferred to soil and cultivated in the greenhouse to confirm the expression of the exogenous *FhMYB5* gene by RT-PCR.

### Quantitative Real-Time PCR Analysis

Specific quantitative real-time PCR primers were designed to detect the spatial and temporal expression patterns of *FhMYB5*. Meanwhile, primers for anthocyanin biosynthetic genes from tobacco and *Arabidopsis* were also designed to detect gene expression levels by quantitative real-time PCR (Supplementary Table S1). RT-qPCR assays were carried out in 20 μL reactions with SYBR Green Mixture (US Everbright^®^ Inc., Suzhou, China) using an ABI7500 RT-qPCR system. Each PCR mixture contained 1 μL of cDNA, 10 μL of 2 × Taq DNA polymerase premix and each primer at 0.25 μM. The reaction mixtures were heated to 95°C for 5 min, 45 cycles at 95°C for 10 s, 60°C for 10 s and 72°C for 20 s. The relative transcript levels were normalized by *Freesia 18S rRNA*, tobacco *Tubulin* and *Arabidopsis Actin*, respectively, and calculated using the 2^-ΔΔCT^ analysis method (Livak and Schmittgen, 2001). All biological replicates were analyzed in triplicate.

### Constructs

Vectors used in this study were constructed using Minerva Super Fusion Cloning Kit (US Everbright^®^ Inc., Suzhou, China). The modified pUC19 vectors with HA, GD, GFP(N) or GFP(C) tag, which were driven by double *35S* promoter of *CaMV* and terminated by *NOS* terminator, were all linearized by *Nde*I and *Afl*II (Wang et al., 2015). To generate constructs for plant transformation, the complete coding sequence of *FhMYB5* cDNA was amplified with specific primers designed to introduce 15-bp overlap regions with the digested pUC19 vector and assembled by Minerva Super Fusion Cloning Kit, creating the pUC19-HA-FhMYB5 plasmid. The pUC19-HA-FhMYB5 vector was then digested by *Eco*RI and the fragments containing *35S* promoter, *HA* tag, *FhMYB5* and *NOS* terminator were cloned into binary vector pPZP211 by T4 DNA ligase (TransGen Biotech, Beijing, China), creating pPZP211-HA-FhMYB5 plasmid, which was then introduced into *Agrobacterium tumefaciens* GV3101 strain before plant transformation. For plasmids used in plant protoplast transient transfection assays, corresponding sequence of each gene was constructed in the backbone of pUC19 according to the protocol mentioned above to generate vectors such as pUC19-GFP(N)-FhMYB5, pUC19-GFP(C)-FhTT8L, pUC19-GFP(C)-FhGL3L, pUC19-HA-AtTT2, pUC19-GD-NtAN1 and pUC19-GD-NtJAF13. As for GUS reporter plasmids used in transient transfection assays, the former constructed AtDFR-pro:GUS vector used in earlier studies was digested by *Pst*I and *Sac*I (Li et al., 2017). The -1,500- to -1000-bp upstreams of *Freesia* anthocyanin biosynthetic genes from the initiation codon “ATG” were assembled into the digested GUS vector following the methods aforementioned. All the other constructs used in this study have been described previously (Li et al., 2016, 2017).

### Transient Transfection Assays

All the plasmids used in transient transfection assays were extracted from *Escherichia coli* using GoldHi EndoFree Plasmid Maxi Kit (CWBIO, Beijing, China) and then concentrated by isopropanol and 5 M NaCl solution. The volume ratio between plasmids, isopropanol and NaCl was 1:1.42:0.42. The mixture was then incubated at room temperature for 10 min followed by centrifugation at 4,000 rpm for 20 min. The pellet was washed at least once by 75% ethanol before dissolved by moderate ultrapure water. The enriched plasmids were subjected to the further transfection assays.

Protoplasts isolation and transfection assays were performed as described in earlier studies (Yoo et al., 2007; Zhou et al., 2014; Li et al., 2016, 2017). Briefly, 3- to 4-week-old *Arabidopsis* leaves were employed for the protoplasts isolation, while 3-week-old subcultured calli were collected to isolate protoplasts from *Freesia*. Different combinations of 10 μg aliquot of effector and reporter plasmids were transfected into protoplasts by PEG3350. After 20–22 h incubation at 22°C in the dark, the transfected protoplasts were gathered by centrifugation and subjected to GUS activity detection using a Synergy^TM^ HT microplate reader (BioTEK, United States) or total RNA extraction with the OminiPlant RNA Kit (CWBIO, Beijing, China) for gene expression analysis. The transient transfection assays were repeated in triplicate with three biological replicates.

### Anthocyanin and Proanthocyanidin Analysis

Total anthocyanin and PA contents were determined in both transgenic and control plants as described in previous study (Li et al., 2016). For anthocyanin measurement, 0.1–0.15 g of each sample was ground into powder in liquid nitrogen and soaked in 1 mL acidic methanol (containing 1% HCl) at 4°C overnight. The solution was centrifuged at 12,000 rpm for 1 min to collect the supernatant. 400 μL of supernatant was mixed with 600 μL of acidic methanol. The absorbance of each sample was measured at 530 and 657 nm using a spectrophotometer (BioTEK, United States), respectively. The relative content of anthocyanin was quantified as (A_530_–0.25 × A_657_) g^-1^ fresh weight (FW). For measurement of PA, 0.1–0.15 g of samples were extracted with 1 mL of 70% (V/V) aqueous acetone solution containing 0.1% (W/V) ascorbic acid at 4°C for 1 h. After centrifugation at 12,000 rpm for 5 min, the supernatant was transferred to a new tube and the precipitate was soaked in another 1 mL of 70% (V/V) aqueous acetone solution containing 0.1% (W/V) ascorbic acid at 4°C overnight. The 2 mL of supernatant was mixed with 1.5 mL of diethyl ether and kept at -20°C in the dark for the separation of two phases. ∼330 μL of lower phase was mixed with 84 μL of DMACA (dimethylaminocinnamaldehyde) regent and 165 μL of methanol, kept at room temperature for 20 min to detect the soluble PA. The DMACA reagent containing 2% (w/v) DMACA in a cold mixture of methanol and 6 M HCl (1:1, v/v) was prepared immediately before use. The PA content was quantified as A_640_ g^-1^ fresh weight (FW). For catechin and epicatechin analysis, 0.1 g of samples were processed as the method of PA measurement aforementioned, the ∼400 μL of lower phase after extraction with diethyl ether was concentrated for 1 h using Vacuum Centrifugal Evaporator (CVE-2000, EYELA, Tokyo, Japan). The concentrated solution was injected through 0.22 μm filtrum and analyzed using an ACCHROM XUnion C18 column (250 mm × 4.6 mm, 5 mm). The column was maintained at 30°C and eluted with solvent A (0.1% phosphoric acid in H_2_O) and B (acetonitrile) following the conditions: 0–16 min, 5–10% B; 16–23 min, 10–13% B; 23–43 min, 13–17% B; 43–48 min, 17–22% B; 48–60 min, 22–30% B; 60–65 min, 30–60% B; 65–72 min, 60–100% B; 72–82 min, 100–5% B; 82–100 min, 5% B. The flow rate was set at 1 mL min^-1^. Detection was monitored at 280 nm. The standard catechin and epicatechin were purchased from Sigma Chemical Company (St. Louis, MO, United States). For anthocyanin and PA measurement in *Freesia* protoplasts, nine replicates of protoplasts transfected by the same combination of constructs were gathered by centrifugation and extracted by acidic methanol or acetone solution aforementioned, respectively. The relative contents of anthocyanin and PA were quantified as A_530_–0.25 × A_657_ or A_640_, respectively. All biological replicates were analyzed in triplicate.

The histochemical staining of PA in tobacco flowers was conducted following the earlier studies with some modifications (Harborne, 1989). The tissues were stained with 0.3 % (w/v) DMACA dissolved in a solution of ethanol: 6 M HCl (1:1) for 30 min, and then washed by 70% ethanol.

## Results

### Isolation and Characterization of *FhMYB5* From *Freesia hybrida*

Initially, the amino acid sequence of *Vitis* VvMYB5a was used as a query to search homologous sequences participating in flavonoid biosynthesis in *Freesia* by mining the *Freesia* transcriptome database through *in situ* TBLASTN program. Consequently, one sequence was seemed to be a general pathway regulator and tentatively named as *FhMYB5* for further functional identification. This 888-bp open reading frame of *FhMYB5* encodes a protein of 295 amino acid residues with a predicted molecular mass of ∼33 kD (Supplementary Table S2).

**FIGURE 1:**
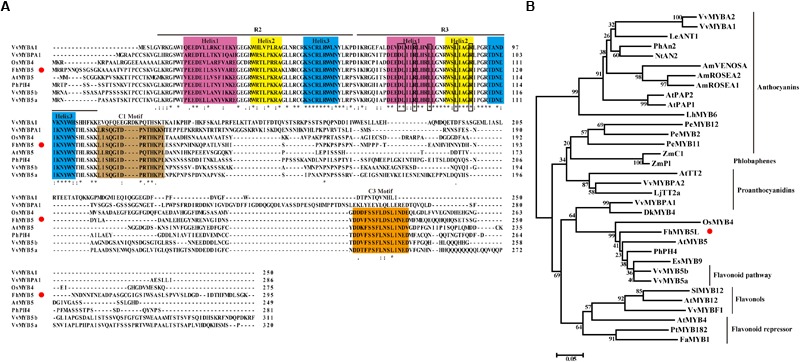
Molecular and phylogenic analysis of FhMYB5. **(A)** Multiple alignment of the full amino acid sequence of FhMYB5 with MYB proteins from other plants. Number indicated the position of the last amino acid in each line. ^∗^, identical amino acids; : or ., similar amino acids. The three helixes in either R2 or R3 repeats were shaded in different colors. Rectangular frames were used to indicate the specific motif [D/E]Lx_2_[R/K]x_3_Lx_6_Lx_3_R responsible for interacting with bHLH factors. The conserved C1 and C3 motifs were also well-marked by colorful backgrounds. Red circle was used for signalizing FhMYB5. **(B)** Phylogenetic analysis of MYB proteins from different plants. The tree was constructed using the neighbor-joining method by the MEGA 6.0 software. The numbers indicated bootstrap values for 1,000 replicates. FhMYB5 was indicated by red circle. The GenBank accession numbers of the MYB proteins were as follows: *Freesia hybrida* FhMYB5 (MK168337); *Antirrhinum majus* AmROSEA1 (ABB83826), AmROSEA2 (ABB83827), AmVENOSA (ABB83828); *Arabidopsis thaliana* AtPAP1 (AAG42001), AtPAP2 (AAG42002), AtTT2 (Q2FJA2), AtMYB12 (CAB09172), AtMYB4 (AAC83582), AtMYB5 (AAC49311); *Vitis vinifera* VvMYBF1 (FJ948477), VvMYB5a (AAS68190), VvMYB5b (Q58QD0), VvMYBPA1 (AM259485), VvMYBPA2 (ACK56131), VvMYBA1 (BAD18977), VvMYBA2 (BAD18978); *Nicotiana tabacum* NtAN2 (ACO52470); *Epimedium sagittatum* EsMYB9 (AFH03061); *Lycopersicon esculentum* LeANT1 (AAQ55181); *Petunia hybrida* PhAn2 (AAF66727), PhPH4 (AAY51377); *Lilium hybrid* LhMYB6 (BAJ05399); *Phalaenopsis equestris* PeMYB2 (KF769467), PeMYB11 (KF769476), PeMYB12 (KF769477); *Zea mays* ZmC1 (AAA33482), ZmP1 (AAA19819); *Lotus japonicus* LjTT2a (BAG12893); *Diospyros kaki* DkMYB4 (BAI49721); *Oryza sativa* OsMYB4 (BAA23340); *Fragaria ananassa* FaMYB1 (AAK84064); *Populus tremula x Populus tremuloides* PtrMYB182 (KP723392); *Solanum lycopersicum* SlMYB12 (ACB46530).

FhMYB5 protein sequence was aligned with other known R2R3-MYB regulators from various plants, which revealed that the deduced FhMYB5 contained the R2R3 imperfect repeats responsible for target DNA binding near its amino-terminal extremity (Figure 1A). The DNA binding domain also contained the conserved motif [D/E]Lx_2_[R/K]x_3_Lx_6_Lx_3_R which was required to interact with bHLH co-factors in R3 domain (Dubos et al., 2010; Liu et al., 2015). Moreover, the alignment also revealed the appearance of conserved peptidic motifs in the C-terminal regions such as C1 (Lx_3_GIDPxTHKPL) which was initially found in MYB subgroup 4 (Kranz et al., 2010). In addition to C1, another conserved motif C3 (DDxF[S/P]SFL[N/D]SLIN[E/D]), whose physiological function has not been characterized yet, was also present in FhMYB5.

In order to better define FhMYB5, a phylogenetic analysis of 33 plant MYB proteins associated with different functions was constructed by the neighbor-joining method and the proteins were clearly defined into different clades according to their functions (Figure 1B). As results, FhMYB5 belonged to none of the clades of proteins specially regulating one branch of flavonoid pathway, but fell within the cluster of *Vitis* VvMYB5a and VvMYB5b which functioned in general flavonoid pathway. In conclusion, sequence analysis indicated that FhMYB5 was a member of R2R3-MYB family, and more precisely, belonged to a small group of MYB family characterized by conserved C1 and C3 motifs which might be involved in general flavonoid biosynthesis.

### Expression of *FhMYB5* Correlates With Anthocyanin and Proanthocyanidin Accumulation in *F. hybrida*

**FIGURE 2:**
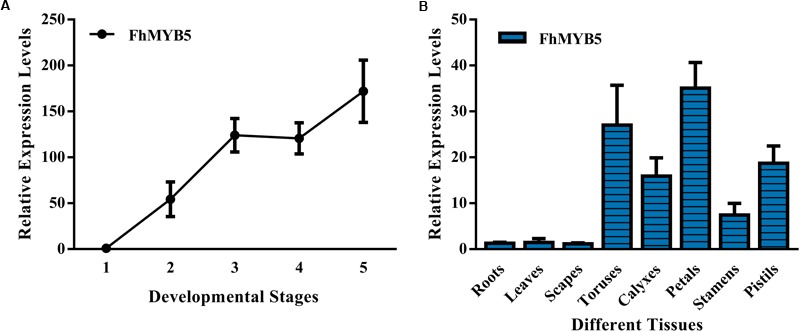
Expression profiles of FhMYB5 in *Freesia hybrida*. **(A)** Relative expression levels of FhMYB5 at five developmental stages. Stage 1, unpigmented buds; Stage 2, slightly pigmented buds; Stage 3, pigmented buds; Stage 4, flowers just before complete opening; Stage 5, fully opened flowers. **(B)** Relative expression levels of FhMYB5 in different tissues. Data represent means ± SD of three biological replicates.

To verify the relationship between the expression levels of *FhMYB5* gene and the flavonoid accumulation, we examined the anthocyanin and PA accumulation together with the expression profiles of the *FhMYB5* gene in five flower developmental stages and eight vegetative or reproductive tissues. As results showed in Supplementary Figure S1 and our earlier studies, anthocyanin levels increased gradually and peaked at blooming flowers, while the abundance of PAs fluctuated at a relative stable level during flower development in the *Freesia* cultivar, Red River^®^ (Li et al., 2016). As for different tissues, anthocyanins and PAs were mainly accumulated in petals and toruses, respectively. RT-qPCR analyses showed that the expression level of *FhMYB5* also increased gradually during the five developmental stages, resembling anthocyanin biosynthesis pattern. Moreover, *FhMYB5* was highly expressed in both petals and toruses, from which we inferred that *FhMYB5* might also be involved in the biosynthesis of other flavonoid compounds other than anthocyanins, such as PAs (Figure 2).

Taken together, these data suggested that the expression of *FhMYB5* was associated with anthocyanin and PA accumulation in *Freesia*. However, the mechanism of how *FhMYB5* controls anthocyanin and PA biosynthesis remains unclear.

### FhMYB5 Is an Activator and May Function With Co-factors

**FIGURE 3:**
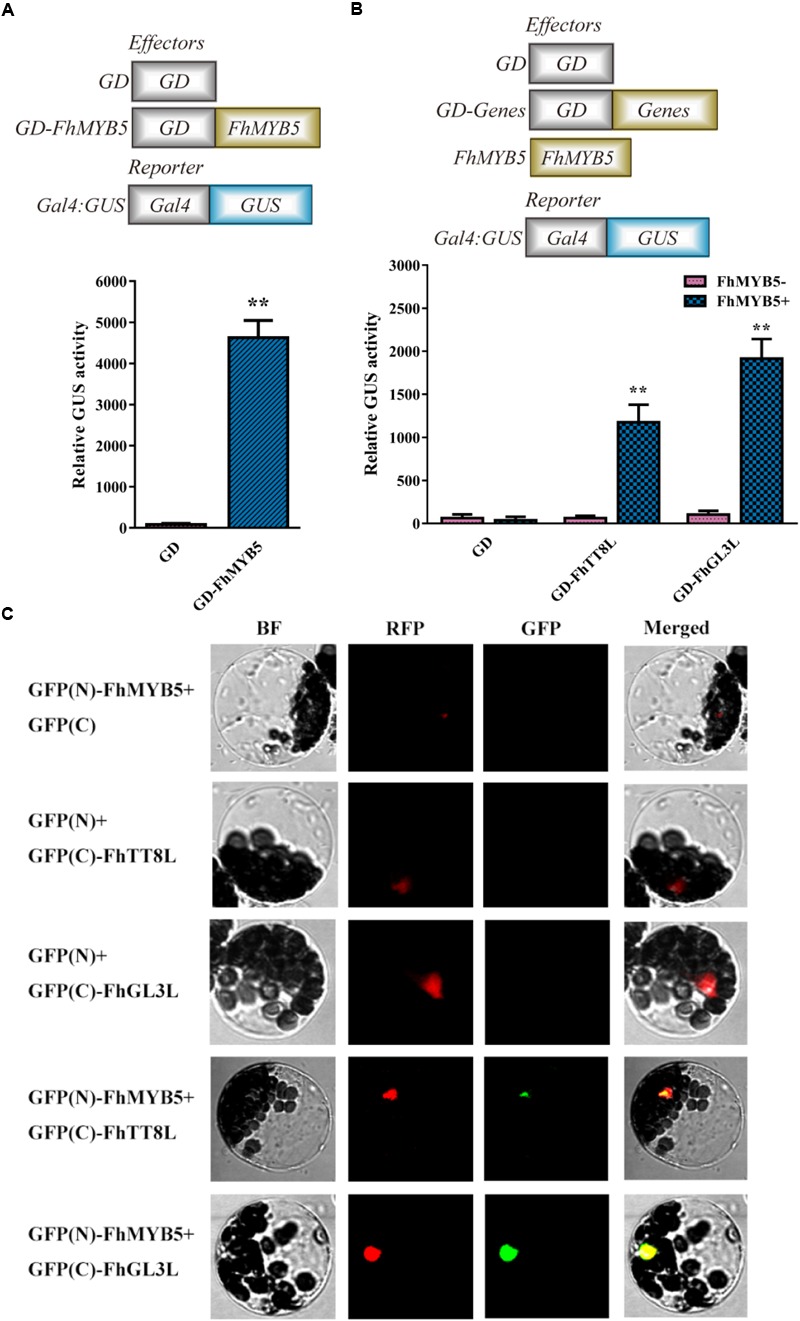
*Trans*-activation ability of FhMYB5 and cooperative interaction between FhMYB5 and bHLH proteins in transient protoplasts experiments. **(A)** FhMYB5 showed high *trans*-activation ability. **(B)** FhMYB5 could interact with FhTT8L and FhGL3L based on the GAL4-GUS system. Different combinations of effector and reporter constructs (diagrammed at the x-axis) were co-transformed into *Freesia* protoplasts. After 20–22 h incubation, the GUS activity was measured. The experiments were replicated at least thrice. Data represented means ± SD of three replicates. *T*-test was carried out to analyze the significant difference (^∗^ represented *P* < 0.05; ^∗∗^ represented *P* < 0.01). **(C)** FhMYB5 could interact with FhTT8L and FhGL3L based on the BiFC experiments. Different combinations of constructs were co-transformed into *Arabidopsis* protoplasts. Moreover, a chimeric red fluorescent protein (RFP) construct containing a nuclear localization signal was included in all the transformation as a positive control. After 20–22 h incubation, the fluorescence was detected. BF, bright field.

Transient protoplast transfection assays were conducted to determine the *trans*-regulation properties of FhMYB5 in *Freesia* cells. As shown in Figure 3A, protoplasts transformed with GD-FhMYB5 effector construct exhibited a drastic increment in beta-glucuronidase activity compared to protoplasts expressing GD only. As a consequence, FhMYB5 could activate reporter gene in protoplasts and thus was confirmed to be an activator with striking *trans*-activation property.

In many model plant systems, it has been established that the combinatorial interactions between MYB and bHLH transcription factors within the MBW complex are crucial for the regulation of the flavonoid pathway (Hichri et al., 2011a; Zhou et al., 2012; Jaakola, 2013; Liu et al., 2015; Xu et al., 2015; Zhao and Tao, 2015). Previously, we have characterized two bHLH regulators regulating anthocyanin and PA biosynthesis in *Freesia* (Li et al., 2016). Thus, a transient protoplasts assay was conducted to assess whether FhMYB5 could interact with FhTT8L or FhGL3L in *Freesia* cells. As shown in Figure 3B, co-transfection of FhMYB5 effector plasmid and GAL4-GUS reporter construct, together with the bHLH protein FhTT8L or FhGL3L, resulted in notable increase of beta-glucuronidase activity, as compared to the control (GAL4-GUS reporter construct with bHLH factor). Moreover, the interactions between FhMYB5 and FhTT8L or FhGL3L were also confirmed by bimolecular fluorescence complementation (BiFC) assays. As shown in Figure 3C, the green fluorescence could only be detected in combinations of FhMYB5 and FhTT8L or FhGL3L. Altogether, these results demonstrated significant interactions between FhMYB5 and FhTT8L or FhGL3L, implying positive roles of FhMYB5 in anthocyanin and PA biosynthesis.

### Overexpression of FhMYB5 in *Freesia* Affects the General Flavonoid Pathway

**FIGURE 4:**
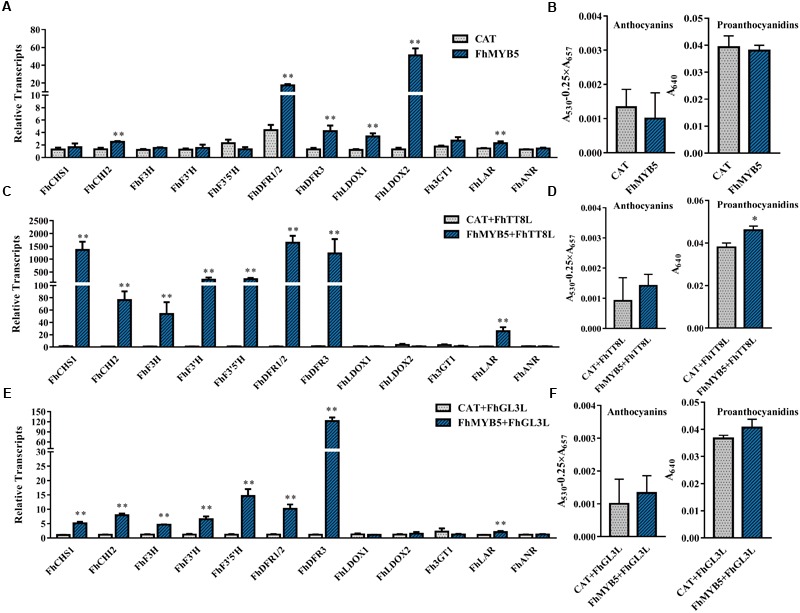
Relative expression levels of flavonoid biosynthetic genes and anthocyanin or PA accumulation in *Freesia* protoplasts overexpressing *FhMYB5*. **(A)** *Freesia* protoplasts isolated from calli tissues were transfected with *FhMYB5*, an irrelevant construct CAT (CHLORAMPHENICOL ACETYLTRANSFERASE) was used as negative control. **(B)** The accumulation of anthocyanin and PA in *Freesia* protoplasts overexpressing *CAT* and *FhMYB5*. **(C)** Relative gene expression levels in *Freesia* protoplasts overexpressing *CAT* and *FhTT8L, FhMYB5*, and *FhTT8L*. **(D)** The accumulation of anthocyanin and PA in *Freesia* protoplasts overexpressing *CAT* and *FhTT8L, FhMYB5*, and *FhTT8L*. **(E)** Relative gene expression levels in *Freesia* protoplasts overexpressing *CAT* and *FhGL3L, FhMYB5*, and *FhGL3L*. **(F)** The accumulation of anthocyanin and PA in *Freesia* protoplasts overexpressing *CAT* and *FhGL3L, FhMYB5*, and *FhGL3L*. The protoplasts were incubated in darkness at room temperature for 20∼22 h, and then harvested for RNA extraction or anthocyanin and PA accumulation. Data represented means ± SD of three replicates. *T*-test was carried out to analyze the significant difference (^∗^ represented *P* < 0.05; ^∗∗^ represented *P* < 0.01).

To identify the potential target genes of FhMYB5 in *Freesia*, we analyzed the ability of the protein to activate *Freesia* genes encoding enzymes of the flavonoid pathway by transient transforming *FhMYB5* alone or in combination with *FhTT8L* or *FhGL3L* together into *Freesia* protoplasts. RNA was extracted from the protoplasts and the expression levels of flavonoid biosynthetic genes were evaluated by RT-qPCR. As results shown in Figure 4, FhMYB5 could only slightly activate *FhCHI* and the late biosynthetic genes including *FhDFR1/2, FhDFR3, FhLDOX1* and *FhLDOX2* when overexpressed alone in *Freesia*. However, the co-expression of *FhMYB5* and *FhbHLH* gene could highly up-regulate the expression of the general flavonoid biosynthetic genes, including *FhCHS, FhCHI, FhF3H, FhF3’H, FhF3’5’H, FhDFR*, and the PA biosynthetic gene *FhLAR* (changing fold was lower compared with other genes). These results suggested that FhMYB5 might function in the common steps of the flavonoid pathway and PA biosynthetic pathway. Moreover, the anthocyanin or PA contents in *Freesia* protoplasts were subsequently detected, a slight but significant increase in PA accumulation was observed when FhMYB5 and FhTT8L were co-transfected (Figure 4D). Nevertheless, the short time transient protoplast transfection assays might not be a strong system to supervise the flavonoid accumulations which need a substantial time to be synthesized.

**FIGURE 5:**
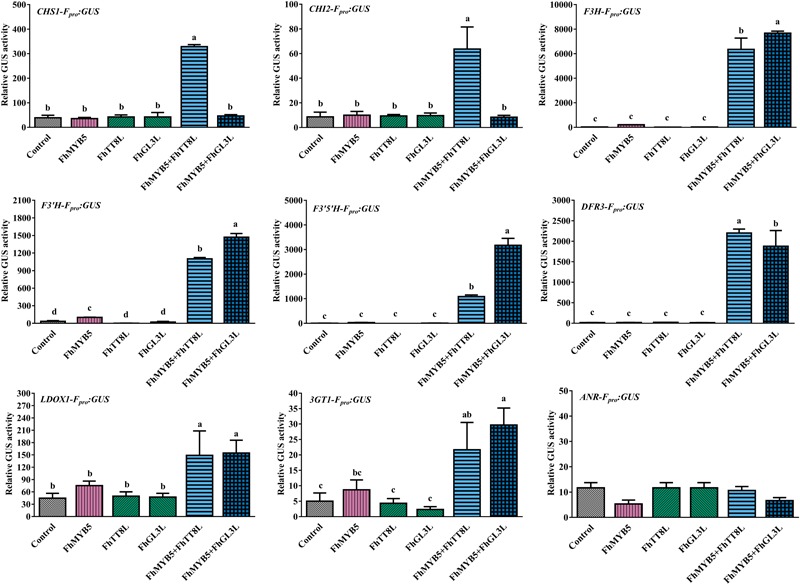
Activation of flavonoid biosynthetic gene promoters by FhMYB5 and FhbHLH regulators. Control indicated the activity of the respective promoter in the absence of FhMYB5 and FhbHLH factors. *FhMYB5* was transfected alone or co-transfected with *FhTT8L* or *FhGL3L* as effectors. The *Arabidopsis* protoplasts were incubated in darkness at room temperature for 20–22 h, and then harvested for GUS activity determinant. Each column represented the mean value of three replicates with error bars indicating SD. One-way ANOVA was carried out to compare statistical differences (Ducan, *P* < 0.05).

To further confirm the ability of FhMYB5 to activate the promoters of *Freesia* flavonoid biosynthetic genes, we constructed the GUS reporter constructs containing the respective promoter of structural genes. Figure 5 summarized the results obtained with different combinations of FhMYB5 and FhbHLH proteins and indicated that no obvious activation effects of FhMYB5 to the flavonoid biosynthetic genes were found in the absence of FhbHLH co-factors. In contrast, significant inductions of the promoters of flavonoid biosynthetic genes, including *FhCHS1, FhCHI2, FhF3H, FhF3’H, FhF3’5’H, FhDFR3* and *FhLDOX1*, were strikingly detected in protoplasts co-transformed with FhMYB5 and FhTT8L or FhGL3L proteins, which was consistent to the RT-PCR analysis mentioned above. In addition, the promoter of *Fh3GT1* could also be slightly activated. Unfortunately, we failed to isolate the promoter of *FhLAR* after numerous attempts and the promoter of *ANR* leading to the synthesis of PAs could not be activated by any combinations. Accordingly, *FhANR* was not highly expressed in PA hyper-accumulation tissues in contrast to *FhLAR* (unpublished data). These findings further indicated that FhMYB5 could mainly enhance the expression of structural genes from the common steps of the flavonoid pathway.

Taken together, gene expression assays and promoter activation data indicated that FhMYB5 could mainly activate the expression of genes encoding enzymes of the general flavonoid pathway involved in synthesis of anthocyanins and PAs.

### Overexpression of *FhMYB5* Impacts on Accumulations of Anthocyanin and Proanthocyanidin in Tobacco and *Arabidopsis*

**FIGURE 6:**
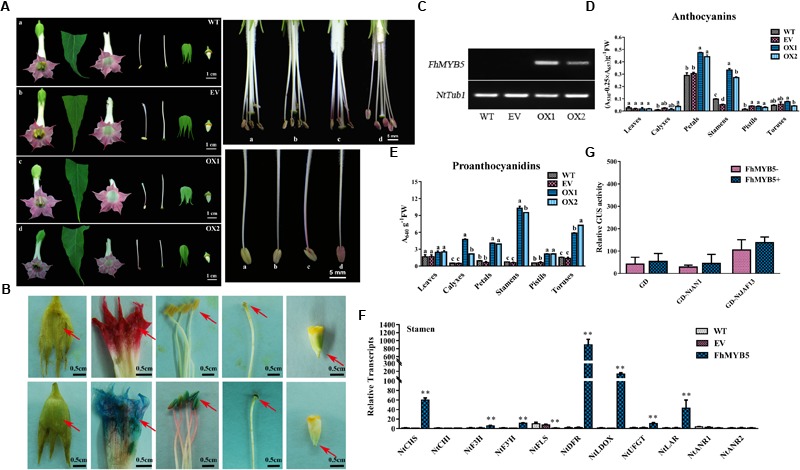
Phenotypic and molecular analysis of transgenic tobacco flowers overexpressing *FhMYB5*. **(A)** Flowers of transgenic plants showed an increased pigmentation in stamen compared to control flowers. WT, wild type plant; EV, plant expressing empty vector; OX, plant overexpressing *FhMYB5*. **(B)** DMACA staining of flower tissues showed proanthocyanidin accumulation when compared to control lines. **(C)** RT-PCR indicated the expression of *FhMYB5* in transgenic tobacco. **(D,E)** The detection of anthocyanins and proanthocyanidins in control and transgenic plants. One-way ANOVA was carried out to confirm statistical differences (Ducan, *P* < 0.05). **(F)** The relative expression levels of flavonoid genes in tobacco stamen detected by RT-qPCR. The relative expression levels of genes were normalized by tobacco *Tubulin*. The assays were repeated three times with three biological replicates. Data represented means ± SD of three replicates. *T*-test was carried out to analyze the significant difference (^∗^ represented *P* < 0.05; ^∗∗^ represented *P* < 0.01). **(G)** Transient transfection assays indicated neither NtAN1 nor NtJAF13 could interact with FhMYB5 in *Arabidopsis* protoplasts. Transformed protoplasts were cultured for 20–22 h at room temperature and harvested for GUS activity determinant. Data represented means ± SD of three replicates. *T*-test was carried out to analyze the significant difference (^∗^ represented *P* < 0.05; ^∗∗^ represented *P* < 0.01).

To ascertain the putative function for *FhMYB5 in planta* and investigate whether it is functionally conserved in angiosperms, we firstly employed tobacco, represents of the Asterids plant, as a model system for heterologous expression experiments. Transgenic tobacco plants overexpressing *FhMYB5* under the control of the cauliflower mosaic virus 35S promoter showed no obvious differences in vegetative tissues compared to the control lines. However, the most important modifications were observed in stamens which were obviously pigmented in transgenic plants (Figure 6A). Moreover, another observation was the hyper-accumulation of PAs in transgenic lines revealed by the DMACA regent which provided a blue staining when interacted with PAs (Figure 6B). In order to confirm whether the phenotypes of transgenic lines resulted from the expression of the exogenous genes, the presence of *FhMYB5* was confirmed by RT-PCR (Figure 6C). Moreover, the relative quantifications of anthocyanin and PA content were accomplished by spectrophotometry detection in different tissues. The most conspicuous increase of anthocyanin content was observed in stamens as expected, while slight increase was also detected in petals (Figure 6D). As for PA accumulation, the increase was almost obvious in all the tissues detected (Figure 6E). Expression analysis performed on RNA extracted from stamens of transgenic and control lines indicated that FhMYB5 could activate the expression of several structural genes associated with the flavonoid pathway, including *NtCHS, NtF3H, NtF3’H, NtDFR, NtLDOX, NtUFGT* and *NtLAR* (Figure 6F). In contrast, the structural genes were differently regulated in other tissues of the transgenic plants. An exception was observed for *NtLAR* which was particularly up-regulated in all the tissues detected (Supplementary Figure S2). As NtLAR synthesized catechins while NtANR synthesized epicatechins, which promoted us to detect whether the PA hyper-accumulation in *FhMYB5* overexpressors was resulted from catechins accumulation, further HPLC assays of the stamens were carried out and results indicated that catechins were significantly synthesized (Supplementary Figure S3). To further decipher the mechanism of FhMYB5 in regulating tobacco flavonoid biosynthesis, we carried out additional analyses on the interaction between FhMYB5 and the earlier-characterized flavonoid biosynthesis related bHLH factors using GAL4-based transient protoplast transfection system aforementioned. Interestingly, neither NtAN1 nor NtJAF13 could interact with FhMYB5 (Figure 6G). Taken together, these results indicated the regulatory mechanisms of FhMYB5 might be different with recently characterized anthocyanin or PA related MYB regulators in tobacco *per se.*

**FIGURE 7:**
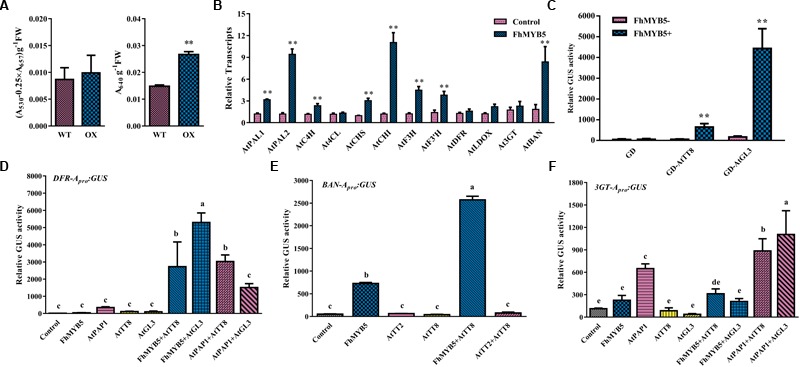
FhMYB5 could interact with *Arabidopsis* bHLH factors in regulating flavonoid biosynthesis related genes. **(A)** The contents of anthocyanins and proanthocyanidins in wild type and transgenic plants. *T*-test was carried out to analyze the significant difference (^∗^ represented *P* < 0.05; ^∗∗^ represented *P* < 0.01). **(B)** Gene expression levels of wild type and transgenic *Arabidopsis* overexpressing *FhMYB5*. Total RNA was extracted from rosette leaves of 4-week-old *Arabidopsis* and RT-qPCR was performed to detect gene expression levels. *T*-test was carried out to analyze the significant difference (^∗^ represented *P* < 0.05; ^∗∗^ represented *P* < 0.01). **(C)** FhMYB5 could interact with AtTT8 and AtGL3. GD tagged bHLH proteins were co-transfected into *Arabidopsis* protoplasts with reporter construct GAL4-GUS in the absence or presence of FhMYB5. *T*-test was carried out to analyze the significant difference (^∗^ represented *P* < 0.05; ^∗∗^ represented *P* < 0.01). **(D–F)** Promoter activation of *AtDFR, AtBAN* and *At3GT* in *Arabidopsis* protoplasts expressing different combinations of effector and reporter constructs. The promoter activation abilities were quantified by relative GUS activities. One-way ANOVA was carried out to compare statistical differences (Ducan, *P* < 0.05). All the transformed protoplasts were cultured at room temperature for 20–22 h in the darkness. Data represented means ± SD of three replicates.

In addition, to further confirm the regulatory function of FhMYB5 in Rosids plants, *Arabidopsis* was selected to perform the transformation assays. However, no obvious phenotype was observed yet. About 4-week-old *Arabidopsis* leaves of T1 progeny were selected for measuring anthocyanin and PA content and the results showed that leaves of *FhMYB5* overexpressors accumulated more PAs than the control (Figure 7A). Raised transcript levels of flavonoid biosynthetic genes, e.g., *AtPAL1, AtPAL2, AtC4H, AtCHS, AtCHI, AtF3H, AtF3’H* and *AtBAN* were revealed by RT-qPCR, implying FhMYB5 had an extensive effect on both *EBGs* and *LBGs* (Figure 7B). In addition, significant interactions between FhMYB5 and *Arabidopsis* bHLH proteins were observed (Figure 7C). We also analyzed the ability of FhMYB5 to activate heterologous promoters using the transient expression assay aforementioned. Resembling the anthocyanin-specific AtPAP1, FhMYB5 is able to activate *AtDFR* at the presence of bHLH proteins (Figure 7D). In addition, it could also dramatically activate *AtBAN* promoter either alone or co-transfected with AtTT8 (Figure 7E). However, FhMYB5 appeared to play subordinate roles in the control of the *At3GT* promoter where the level of activation was very low (Figure 7F). These results reinforced the preliminary conclusion that FhMYB5 appeared to implicate in the control of general flavonoid biosynthesis in *Freesia* and functioned when heterologously expressed in other plants.

## Discussion

### FhMYB5 Belongs to VvMYB5b Subclade

Since the first MYB regulator COLORED1 (C1) was identified in the aleurone of maize kernels, a tremendous amount of MYB proteins have been investigated in numerous plant species such as *Arabidopsis thaliana, Zea mays, Petunia hybrida, Oryza sativa, Antirrhinum majus, Populus tremuloides, Vitis vinifera* L., and *Malus domestica*, etc. Four major MYB groups have been recognized based on the number and placement of MYB domains and the R2R3-MYB proteins have been further classified into 28 subgroups according to the conserved amino acid sequence motifs in their C terminus (Stracke et al., 2001; Dubos et al., 2010). The extensive functional characterizations demonstrated that most MYB proteins played central roles in plant-specific processes as a member of regulatory networks controlling development, primary and secondary metabolism and being in response to biotic and abiotic stresses (Dubos et al., 2010).

In this study, we firstly isolated a flavonoid biosynthesis related R2R3-MYB regulator in *F. hybrida*, designated as *FhMYB5*, whose protein sequence displayed 68% similarity to AtPAP1. The high levels of similarity were relevant considering the significant similarities within R2R3 domain (Supplementary Table S2). Protein structure and phylogenic analysis could help predict functions of the new found MYB, as primary protein structures and biological functions were usually clustered within the same subgroups. Firstly, the existence of the conserved motif [D/E]Lx_2_[R/K]x_3_Lx_6_Lx_3_R implied that FhMYB5 might have the ability to interact with bHLH regulators. This was further validated by transient protoplast assay in our study (Figure 3). Actually, it seems that the interaction was indispensable for FhMYB5 in activating the promoters of *Freesia* genes (Figure 5). Secondly, the conserved C1 motif (Lx_3_GIDPxTHKPL) closely related to amino acid residues, which were found in the maize transcriptional activator C1, could also be found in FhMYB5. The C1 motif is common in MYB subgroup 4, most of which tend to be negative regulators of phenylpropanoid-derived compound synthesis (Pérez-Díaz et al., 2015; Yoshida et al., 2015). Considering the existence of C1 motif, we arbitrarily regarded FhMYB5 as a phenylpropanoid-related suppressor. However, *trans*-activation and genetic transformation assays verified that FhMYB5 turned out to be a flavonoid biosynthesis related activator. Moreover, FhMYB5 also contained the conserved motif C3 (DDxF[S/P]SFL[N/D]SLIN[E/D]) which only appeared in VvMYB5b cluster including AtMYB5, OsMYB4, PH4, BNLGHi233, EsMYB9, VvMYB5a and VvMYB5b as described by Deluc et al. (2006; 2008; Huang et al., 2012, 2017). Functionally, the proteins in this cluster seemed to be involved in the control of various processes like cell fate determination in *Arabidopsis* for AtMYB5 (Li et al., 2009), chilling tolerance in *Oryza sativa* for OsMYB4 (Vannini et al., 2010), vacuolar acidification in *Petunia hybrida* for PH4 (Quattrocchio et al., 2006), anthocyanin and flavonol biosynthesis in *Epimedium sagittatum* for EsMYB9 (Huang et al., 2017), and phenylpropanoid pathway in *Vitis vinifera* for VvMYB5a and VvMYB5b (Deluc et al., 2006, 2008). Additional data were also provided to indicate the functional conservation between FhMYB5 and VvMYB5b, such as the appearance of the same phenotypic changes in transgenic plants, activation of flavonoid biosynthetic genes, the accumulation of anthocyanin and PA-derived compounds and the promoter activation capacity to induce promoters of the general flavonoid pathway genes. Thus, these functional characterization data reinforced that FhMYB5 was a new member of the VvMYB5b cluster (Figure 1).

### FhMYB5 Is Mainly Responsible for the General Flavonoid Genes to Regulate the Biosynthesis of Anthocyanin and Proanthocyanidin

The expression levels of *FhMYB5* increased gradually during *Freesia* flower development and the highest level was detected in petals, which correlated well with anthocyanin accumulation (Li et al., 2016). When overexpressed in *Freesia* protoplasts, FhMYB5 could highly activate most of flavonoid biosynthetic genes transiently except the anthocyanin specific structural gene *Fh3GT1* (Figure 4). This could be partly confirmed by the promoter activation experiment which indicated a lower activation of *Fh3GT1* promoter (Figure 5). In grapevine, VvMYB5b was also able to activate several genes of the general pathway, but not the anthocyanin specific *UFGT* in promoter activation experiments in grape cells (Deluc et al., 2008). In addition to the anthocyanin accumulated tissues, e.g., petal, *FhMYB5* was also found to be highly expressed in toruses where PAs were hyper-accumulated, indicating a positive correlation between *FhMYB5* and PAs (Figure 2). Generally in plants, PAs are condensed from two kinds of monomers, catechins synthesized by LAR and epicatechin synthesized by ANR (Xie et al., 2003; Bogs et al., 2005; Tsai et al., 2006). The homologs of *LAR* and *ANR* were also isolated from *Freesia* (unpublished data). Transient expression assays indicated that FhMYB5 could only activate *FhLAR* expression in *Freesia* cells. However, the increase fold was also significantly lower when compared with the general flavonoid genes. Similar results were also observed in earlier studies about VvMYB5a and VvMYB5b. Though VvMYB5b could activate the expression of *VvLAR* and *VvANR*, and VvMYB5a appeared only implicated in *VvLAR* regulation, the activation levels were low when compared with general flavonoid genes (Deluc et al., 2008). Altogether, it might be reasonable to deduce that FhMYB5 could play roles in the regulation of both anthocyanins and PAs. More precisely, considering the differences of activation levels, FhMYB5 is mainly responsible for the regulation of the general flavonoid genes.

Recently, Uwagaki et al. (2015) has successfully established a method for Agrobacterium-mediated transformation of cormlet-derived calli of *F. hybrida*. Unfortunately, it usually took 18–24 months from inoculation to generate flowered plantlets and the regenerate rate was low. As the MYB proteins within a given subgroup were quite well conserved across angiosperm species, the *in planta* function of FhMYB5 in flavonoid biosynthesis was herein further validated in tobacco and *Arabidopsis*, respectively. As expected, FhMYB5 could mainly induce expression of the general flavonoid genes. Moreover, the gene expression analysis in transgenic tissues of tobacco further consolidated the fact that *NtLAR* is preferentially the potential target gene of FhMYB5 as *NtLAR* is up-regulated in all the transgenic tissues (Figure 6 and Supplementary Figure S1). Further analysis of catechin and epicatechin contents also consolidated that FhMYB5 mainly promoted catechin accumulation in tobacco stamens (Supplementary Figure S3). However, as lack of *LAR* homologs in *Arabidopsis*, FhMYB5 turned to activate *AtBAN* expression in transgenic plants or protoplasts alternatively (Figure 7). In addition, previous studies revealed that the *AtBAN* promoter could hardly be activated unless the co-expression of *AtTT2, AtTT8*, and *AtTTG1* (Baudry et al., 2004). Unexpectedly, FhMYB5 could significantly activate *AtBAN* either alone or co-expressed with AtTT8, implying a stronger role of FhMYB5 in PA biosynthesis of *Arabidopsis*. However, there might be other MYB factors participating in *Freesia* anthocyanin and PA synthesis as *Fh3GT1* and *FhANR* could hardly be activated by FhMYB5. Similarly, besides VvMYB5a and VvMYB5b in grapevine, there were also other MYB regulators including anthocyanin specific VvMYBA1 and VvMYBA2, PA specific VvMYBPA1 and VvMYBPA2, working coordinately on anthocyanin and PA regulation (Kobayashi et al., 2002, 2004; Bogs et al., 2007; Terrier et al., 2009). Actually, we have also cloned three PAP1-like and four TT2-like transcription factors in *Freesia* but their roles remain to be further unveiled (unpublished data).

### The Target Genes of FhMYB5 May Be Affected by Different Co-factors

The ternary complex composed of MYB and bHLH transcription factors, together with WD40 proteins (designated as MBW complex) has been extensively elucidated, and the MYB proteins play critical roles in the determination of *cis*-elements and thus contribute to the selection of target genes (Hartmann et al., 2005; Park et al., 2010; Jaakola, 2013; Xu et al., 2015). However, more and more studies indicated that the affinity between MYB proteins and *cis*-element might partly depend on the nature of co-factors.

The results presented in this study indicated that a single gene *FhMYB5* might impact not only on one branch of the flavonoid pathway, but on nearly the whole pathway. However, unlike other MYB regulators such as AtPAP1 in *Arabidopsis*, which appeared to function on the same targets in all vegetative and reproductive tissues when overexpressed, the function of FhMYB5 appeared variable when overexpressed in tobacco, depending on the tissue studied (Supplementary Figure S1) (Zhou et al., 2008; Shi and Xie, 2010). And it seemed that other potential co-factors remaining uncharacterized might exist in tobacco as FhMYB5 could regulate flavonoid biosynthesis independent of NtAN1 or NtJAF13 (Figure 6G). Consequently, it is possible that a specific interacting partner might exist in special tissues and the lack of a specific co-factor might explain the phenotypic differences of different tissues. The phenomena were also observed in earlier studies about VvMYB5a and VvMYB5b (Deluc et al., 2006, 2008). Moreover, as shown in Figure 4, the activation of flavonoid biosynthetic genes by FhMYB5 in promoter activation experiments in *Freesia* cells largely depended on the bHLH co-factors. This also substantiated the versatile roles of FhMYB5 *in planta* as it could interact with different co-factors like FhTT8L or FhGL3L or other proteins. Moreover, parameters including cell and tissue type, developmental stage, and environmental conditions could also affect the pattern of MYB regulation. Nevertheless, the functional identification of FhMYB5 in this article underlined the potential of elucidating the mechanisms of flavonoid biosynthesis in *F. hybrida* and the evolutionary conservation of MYB regulators in angiosperm plants.

## Conclusion

In this study, the first MYB regulatory gene *FhMYB5* was isolated and performed as candidate gene implicated in flavonoid biosynthesis in *Freesia* flowers. The results provided substantial proof that FhMYB5 was able to regulate various branches of the flavonoid pathway in *Freesia* protoplast transient transfection system, as well as in tobacco and *Arabidopsis* when overexpressed and seemed mainly to be anthocyanin and PA biosynthesis related regulator. However, compared with the relative low activation levels of *Fh3GT1* and *FhLAR*, the strong expression of genes encoding enzymes of the general flavonoid pathway was found, indicating that FhMYB5 might be involved in the control of the general flavonoid pathway.

## Author Contributions

YL, XS, LZ, RG, and SY performed most of the experiments. SW analyzed the results. XG designed the experiments, wrote and edited the manuscript. LW designed the experiments and revised the manuscript. All authors read and approved the final manuscript.

## Conflict of Interest Statement

The authors declare that the research was conducted in the absence of any commercial or financial relationships that could be construed as a potential conflict of interest.
