# Supplementary material for: The R2R3-MYB Factor FhMYB5 From Freesia hybrida Contributes to the Regulation of Anthocyanin and Proanthocyanidin Biosynthesis
Source: Front Plant Sci. 2019 Jan 7;9:1935. doi: 10.3389/fpls.2018.01935 (PMC6330306; doi:10.3389/fpls.2018.01935)
Supplement: Supplementary file 1 [file Data_Sheet_1.doc]

**The R2R3-MYB Factor FhMYB5 from *Freesia hybrida* Contributes to the Regulation of Anthocyanin and Proanthocyanidin Biosynthesis**

Yueqing Li^†1^, Xiaotong Shan^†1^, Liudi Zhou^1^, Ruifang Gao^1^, Song Yang^1^, Shucai Wang^1^, Li Wang^1*^, Xiang Gao^1, 2*^

^1^ Key Laboratory of Molecular Epigenetics of MOE and Institute of Genetics & Cytology, Northeast Normal University, Changchun, China

^2^ National Demonstration Center for Experimental Biology Education, Northeast Normal University, Changchun, China

*To whom correspondence should be addressed. E-mail address: gaoxiang424@163.com; wanglee57@163.com

Tel.:+86 431 85099360;

^†^ These authors contributed equally to this work

Table S1: Primers used in the study

|  | | Forward(5'-3') | Reverse(5'-3') |
| --- | --- | --- | --- |
| Full length cDNA sequence cloning |  |  |  |
|  | *FhMYB5* | AGAAACAAAGAGAGAATGAGGAGAC | CTTAAACTTACAAGCGATATATATGAG |
|  | *FhCHS1* | GCAAGAAAAATGGTGAATG | TGGGAATGATATATAGGGAGTC |
|  | *FhCHI2* | TTCTGATGACAATGGTGGAG | TTTCTCTCTGCCTCTCCATA |
|  | *FhF3H* | CATCCTGAGACACTAGAGCGA | TCTGCAAATTCTGTAGCAACAC |
|  | *FhF3’H* | ATGCCTACCTTCTTCTTCCT | CACTTCTTCTCATGGGATACA |
|  | *FhF3’5’H* | ACATGGGGAAGCACATAATATG | AGTGGAAGCATATATATATCGA |
|  | *FhDFR2* | GGGGCCTAGGAAAGAACGATGGGGA | GCCCTTATTTCACCTTCCAGCTAGT |
|  | *FhDFR3* | ATGGCCATTACAGCCGACACAGAGA | CACTCCAGGGCACTAATCTCAG |
|  | *FhLDOX1* | GGGAATCATATTTGATCACATA | AGGAAAACAAGCAGGGCATCA |
|  | *FhLDOX2* | ACATGGGGCCCACCATCCTA | GAAAATAAAATATTGGAGCAGG |
|  | *Fh3GT1* | CAGCAAGCAATGGGATCG | CAGACTTCAGTCATATTCCGA |
|  | *FhLAR* | ATGTCTACTCATGAACTCGGCC | TCAGTGAGGTAGATCGGTGGCATAT |
|  | *FhANR* | ATGAGTAGCCCAATCTGTGTTACCG | TTACATCCCCAATCTCTTCATCCAC |
| Transient Luciferase Assay |  |  |  |
|  | *HA-FhMYB5* | CTGATTACGCTCATATGAGGAGACCTAATAAT | AGGATTCAATCTTAAGTTACTTCCCAGAAAGATC |
|  | *GD-FhMYB5* | CTGTATCGCCGCATATGAGGAGACCTAATAAT | AGGATTCAATCTTAAGTTACTTCCCAGAAAGATC |
|  | *GFP(N)-FhMYB5* | ACAACATCGAGGACCATATGAGGAGACCTAATAAT | AGGATTCAATCTTAAGTTACTTCCCAGAAAGATC |
|  | *GFP(C)-FhTT8L* | GACGAGCTGTACAAGCATATGGCGTCGCAGCAGTC | AGGATTCAATCTTAAGCTAGTATTGGGAGAAGAT |
|  | *GFP(C)-FhGL3L* | GACGAGCTGTACAAGCATATGTGTCACTCAATGGAA | AGGATTCAATCTTAAGTCAACATATGCCAACAAC |
|  | *HA-AtTT2* | CTGATTACGCTCATATGGGAAAGAGAGCAACTACT | AGGATTCAATCTTAAGTCAACAAGTGAAGTCTCGGAG |
|  | *GD-NtAN1* | CTGTATCGCCGCATATGACGGAGATACCGCCTAAC | AGGATTCAATCTTAAGTTAATCTCTAGGGATTATCTGAT |
|  | *GD-NtJAF13* | CTGTATCGCCGCATATGGCTATGGGACACCAAGA | AGGATTCAATCTTAAGTCAAGATTTCCAGACTACTCGCT |
|  | *GD-SlAN1* | CTGTATCGCCGCATATGGAGATTATACAGCCTAA | AGGATTCAATCTTAAGTTAATTAACTCTAGGGATTAT |
|  | *GD-SlJAF13* | CTGTATCGCCGCATATGGCTATGGGACACCAAGA | AGGATTCAATCTTAAGTCAAGATTTCCATACTACTC |
| Promoter sequence cloning |  |  |  |
|  | *ProAtDFR* | CCAAGCTTGCATGCCTGCAGGAGATTGGCACCACCTTCGCCTC | CTAAGCTTACCATGAGCTCTTTTGTGGTTATATGATAGATTGTGCT |
|  | *ProFhCHS1* | CCAAGCTTGCATGCCTGCAGATAGTAATATTCGTGTTGGTCGAAG | CTAAGCTTACCATGAGCTCGGCTACAAAATCTTTCTTACA |
|  | *ProFhCHI2* | CCAAGCTTGCATGCCTGCAGTAGGTCAATAATCCCCATTCCATCT | CTAAGCTTACCATGAGCTCCACTTCCATCTCCACCATTGTCAT |
|  | *ProFhF3H* | CCAAGCTTGCATGCCTGCAGTGCATATTGTTGCTCGATGATTTCC | CTAAGCTTACCATGAGCTCTGGCGTTGAAGTCGCGACCGG |
|  | *ProFhF3'H* | CCAAGCTTGCATGCCTGCAGACCATATGTTGAATAATATTGCTTT | CTAAGCTTACCATGAGCTCAATCCAGAGGAAGAAGAAGGTAGGC |
|  | *ProFhF3'5'H* | CCAAGCTTGCATGCCTGCAGTTTGATTATAAATGATAGAAGGAGC | CTAAGCTTACCATGAGCTCATCAAGGGAAATCATTCTCATATTA |
|  | *ProFhLDOX1* | CCAAGCTTGCATGCCTGCAGGTGTATTACTTTAGAGTATT | CTAAGCTTACCATGAGCTCCATCGGCACACTGCTAACCAT |
|  | *ProFhANR* | CCAAGCTTGCATGCCTGCAGCTCCTGCCCTGAAAATC | CTAAGCTTACCATGAGCTCGATTGGGCTACTCATCTT |
|  | *ProFh3GT1* | CCAAGCTTGCATGCCTGCAGTTGATCAGATTCTCATTATT | CTAAGCTTACCATGAGCTCGGAGCGATCGGCCGATCCCAT |
| qRT-PCR |  |  |  |
|  | *FhMYB5* | CTGGATGAATTATCTGCGG | CCAAGAAGTCTGTGGAGCCT |
|  | *FhCHS1* | GGACAGGCATTGTTTGGTG | GAGAAGGTGGAAAGTGAGG |
|  | *FhCHI2* | CAATGGTGGAGATGGAAGTGT | GTGAGGTGAGGTTGGAAGAGA |
|  | *FhF3H* | GGTTCATTGTCTCCAGCCAC | GCTTTTCGCTGTATGTCTTCGT |
|  | *FhF3’h* | GCACAAAGCCTCACCAGAC | TAGGGGGCGAAAACCAAGT |
|  | *FhF3’5’h* | TGGTGGTGGAGGTGATGACT | CCTTGAATGTCGAACGGCT |
|  | *FhDFR2* | GACTGGCTGGATGTATTTTGTG | TGATCATACTTGGTGGCATAG |
|  | *FhDFR3* | CATTAGTTGTCGGTCCTTTCAT | AAGTTGCGCTTGCTTTATTAT |
|  | *FhLDOX1* | AGGAGCAGTATGCGAACAACC | CTCCCACTCCAACTGCCCAC |
|  | *FhLDOX2* | GAGGTCAAGAAGTCGGAGGA | CCCGTGGTTCACAATATGC |
|  | *Fh3GT1* | ACTGCGGATGGAACTCTGT | TTTCCAAACATGCGACACG |
|  | *FhLAR* | CCCATGACATCTTCATAAACGG | TCTCAGGGTAGAGGGAACTCAC |
|  | *FhANR* | ACGAATCATCTTGCGTTTGTGC | GTTCCTGGCTGTGAGCAAATC |
|  | *18S rRNA* | TCCTGATACGGGGAGGTAGTGACA | ACTTGCCCTCCAATGGATCCTCG |
|  | *AtPAL1* | CTTGGAACAGAGCTTTTGACCG | CGTGAAAACCTTGTCGAACTCTTC |
|  | *AtPAL2* | ACCGGCCGTCCTAATTCC | GCTTCTTTCGCGGTTAGCGATTC |
|  | *AtC4H* | TCTCCTCGTGCCTCACATGA | TGCTTTCTGCTGGGATATCGTA |
|  | *At4CL* | GCCACTAAGCCTTGCCTAATCA | CGTGGACGTCGGAGTAAGTGT |
|  | *AtCHS* | GGCAAAGAAGCGGCAGTGAAG | CGGAAGGACGGAGACCAAGAAG |
|  | *AtCHI* | CTCTCTTACGGTTGCGTTTTCG | CACCGTTCTTCCCGATGATAGA |
|  | *AtF3H* | GACCAAGTCGGTGGATTACAAGC | TCCTTCAACAGGCTGAACCG |
|  | *AtF3’H* | TTCCTTACCTTCAGGCGGTTATC | CGAGAGTGGTGTTGGTGGATG |
|  | *AtDFR* | CTTTGTTCGTGCCACCGTTCG | TCCTTCCTCAGATAAATCAGCCTTCC |
|  | *AtLDOX* | GTTTGCAGCTTTTCTACGAGG | TGAGCAAAAGTCCGTGGAGG |
|  | *At3GT* | TGTCAGATCGTTTTGGTTCC | GATTCTTCCTCACTTTCTCAC |
|  | *AtBAN* | AACAACTAAATCTCTATCTCTGTA | GAATGAGACCAAAGACTCATATAC |
|  | *AtACTIN* | GCTGAGAGATTCAGATGCCCA | GTGGATTCCAGCAGCTTCCAT |
|  | *NtCHS* | AGCGAGCATAAGGTTGAG | ACCACCACTATGTCTTGTC |
|  | *NtCHI* | GAAATCCTCCGATCCAGTGA | CAACGTTGACAACATCAGGC |
|  | *NtF3H* | ACAGGGTGAAGTGGTCCAAG | CCTTGGTTAAGGCCTCCTTC |
|  | *NtF3’H* | TCCAAGAATACTGGCCCAAG | CTCACAACTCTCGGATGCAA |
|  | *NtDFR* | TAAGAAGATGACAGGATGGATG | TGGCGGTATGATGCTAATG |
|  | *NtLDOX* | TGGCGTTGAAGCTCATACTG | TTTCAAGGGTGTCCCCAATA |
|  | *NtUFGT* | GAGTGCATTGGATGCCTTTT | CCAGCTCCATTAGGTCCTTG |
|  | *NtLAR* | TCAAGGTCCTTTACGCCATC | ACGAACCTGCTTCTCTTTGG |
|  | *NtANR1* | CATTTGACTTTCCCAAACGC | ATTGGGCTTTTGAGTTGTGC |
|  | *NtANR2* | TGTTCCCACTTGGGATGATA | TGCACCTATACTCTGTTAGTGGC |
|  | *NtTub1* | TCCGTGGTGATGTTGTG | TGGTGGCTGATAGTTGATAC |

TableS2. Information of anthocyanin and proanthocyanidin biosynthetic genes

| **Candidate transcripts** | **Protein sequence length** | **Top *Arabidopsis* BLAST match** | **Top BLAST match excluding *Arabidopsis*** | **Homology (%)** | **Gene bank number of the homology genes used in Blast** |
| --- | --- | --- | --- | --- | --- |
| Structure genes | | | | | |
| *FhCHS1* | 389aa | NP_196897.1 *CHS Arabidopsis thaliana* | AEO45114.1 *CHS Freesia hybrid cultivar* | 84^a^,100^b^ | AEO45114.1 |
| *FhCHI2* | 256aa | NP_191072.1 *CHI* *Arabidopsis thaliana* | AFM36773.1 *CHI Narcissus tazetta var. chinensis* | 62^a^,69^b^ | AHL83557.1 |
| *FhF3H* | 419aa | Q9S818.1 *F3H* *Arabidopsis thaliana* | BAM28970.1 *F3H*  Lilium hybrid division I | 73^a^,73^b^ | BAF44477.1 |
| *FhF3’H* | 467aa | Q9SD85.1 F3’H *Arabidopsis thaliana* | AHL83556.1 *F3’H Iris x hollandica* | 59^a^,80^b^ | AHL83556.1 |
| *FhF3’5’H* | 528aa | - | *AHX02955.1 F3’5’H* Iris x hollandica | 74^b^ | AEB96145.1 |
| *FhDFR2* | 349aa | P51102.2 *DFR* *Arabidopsis thaliana* | BAF93856.1 *DFR* *Iris x hollandica* | 65^a^,79^b^ | BAF93856.1 |
| *FhDFR3* | 358aa | P51102.2 *DFR* *Arabidopsis thaliana* | BAF93856.1 *DFR* *Iris x hollandica* | 65^a^,77^b^ |
| *FhLDOX1* | 361aa | Q96323.1 *LDOX* *Arabidopsis thaliana* | BAF62629.1 *ANS Iris x hollandica* | 65^a^,77^b^ | BAF62629.1 |
| *FhLDOX2* | 366aa | Q96323.1 *LDOX* *Arabidopsis thaliana* | BAF62629.1 *ANS Iris x hollandica* | 67^a^,85^b^ |
| *Fh3GT1* | 446aa | NP_197207.1 *3GT Arabidopsis thaliana* | ADK75021.1 *3GT Freesia hybrid cultivar* | 45^a^,100^b^ | ADK75021.1 |
| *FhLAR* | 322aa | - | *XP_010926155.1 LAR-like* *Elaeis guineensis* | 73^b^ | AFW59563.1 |
| *FhANR* | 338aa | AF092912.1 *BAN*  *Arabidopsis thaliana* | XP_010915680.1 *ANR-like Elaeis guineensis* | 31^a^,60^b^ | NC_003070.9 |
| MYB factors | | | | | |
| *FhMYB5* | 296 residues | NP_176057.1 *MYB75*  *Arabidopsis thaliana* | XP_009421291.1 PREDICTED: transcription repressor MYB5-like Musa acuminata subsp. malaccensis | 68^a^,52^b^ | NP_176057.1 |

^a^% Similarity to *Arabidopsis*.

^b^% Similarity to sequences from other plant species.


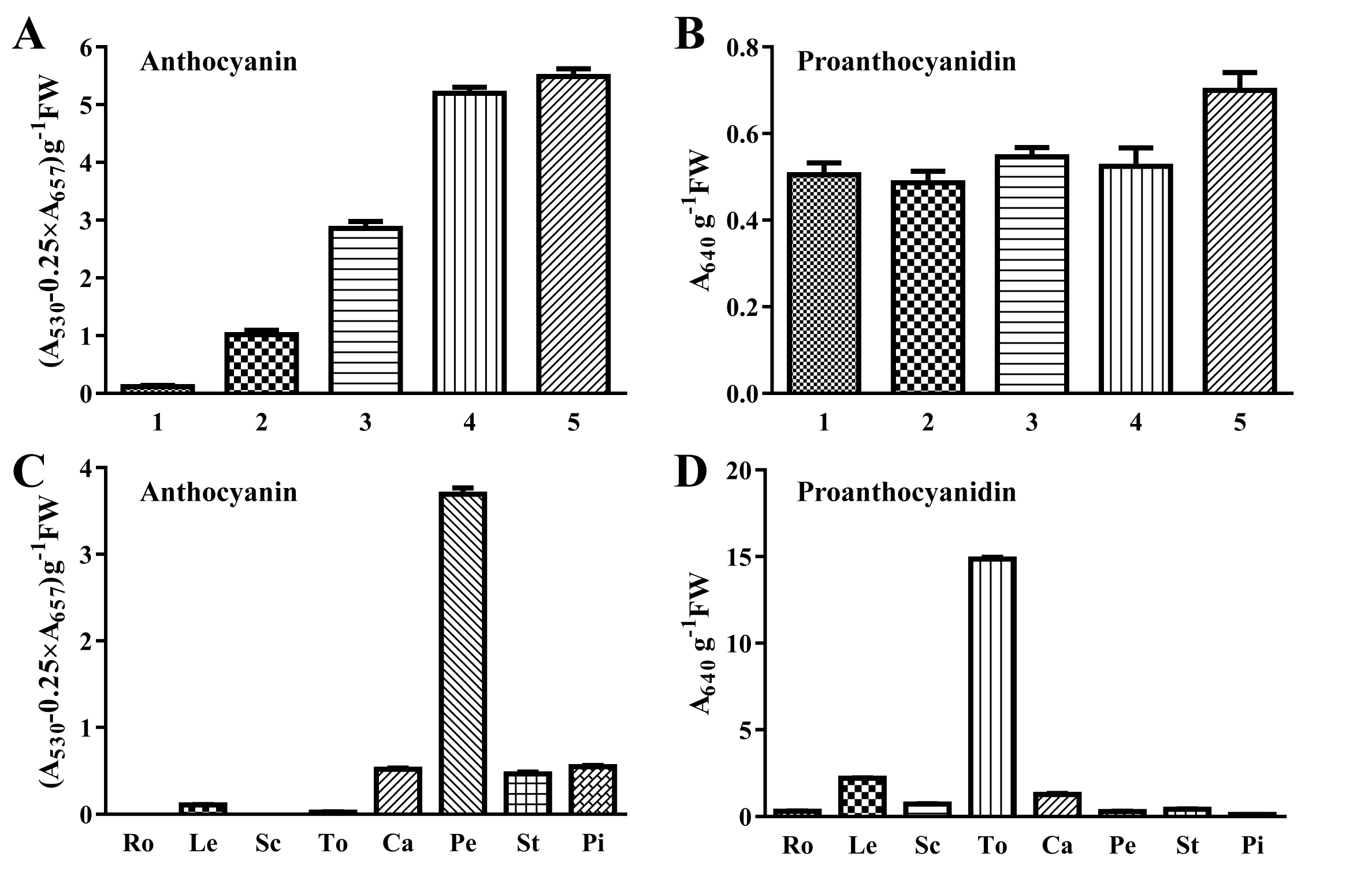


Figure S1. The *Freesia* anthocyanin and PA accumulation profiles in five flower developmental stages and eight vegetative or reproductive tissues. (A) and (C), the anthocyanin accumulation at different developmental stages and in different tissues. (B) and (D), the proanthocyanidin accumulation at different developmental stages and in different tissues. Data represented means ± SD of three replicates. 1-5, represented the flowers of different developmental stages. Ro, roots; Le, leaves; Sc, scapes; To, toruses; Ca, calyxes; Pe, petals; St, stamens; Pi, pistils. FW, fresh weight.


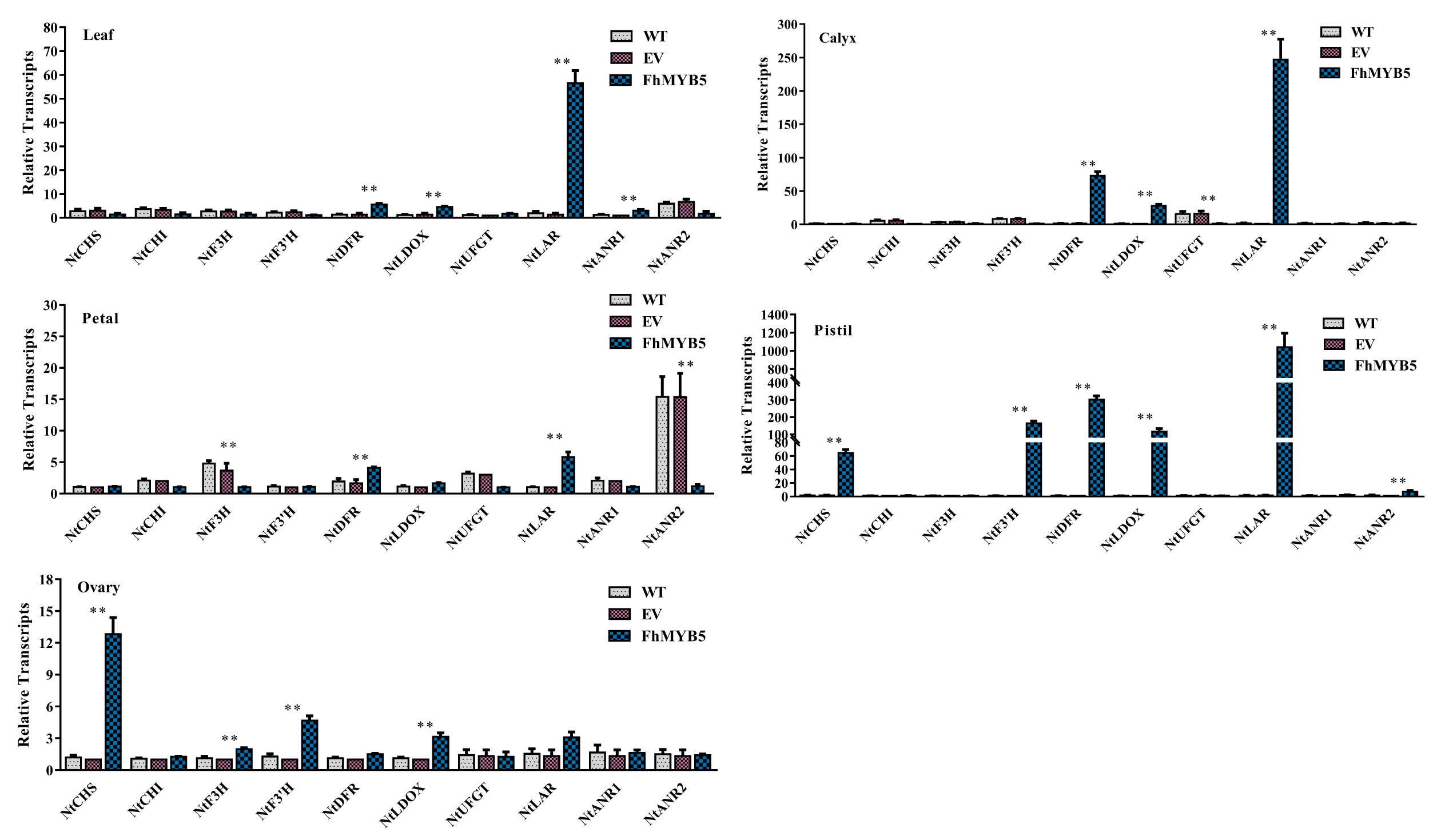


Figure S2. The relative expression levels of flavonoid biosynthetic genes in tobacco tissues detected by RT-qPCR. The relative expression levels of genes were normalized by tobacco *Tubulin*. Data represented means ± SD of three replicates. T-test was carried out to analyze the significant difference (* represented P < 0.05; ** represented P < 0.01). WT, wild type tissues; EV, tissues expressing empty vectors; FhMYB5, tissues overexpressing FhMYB5.


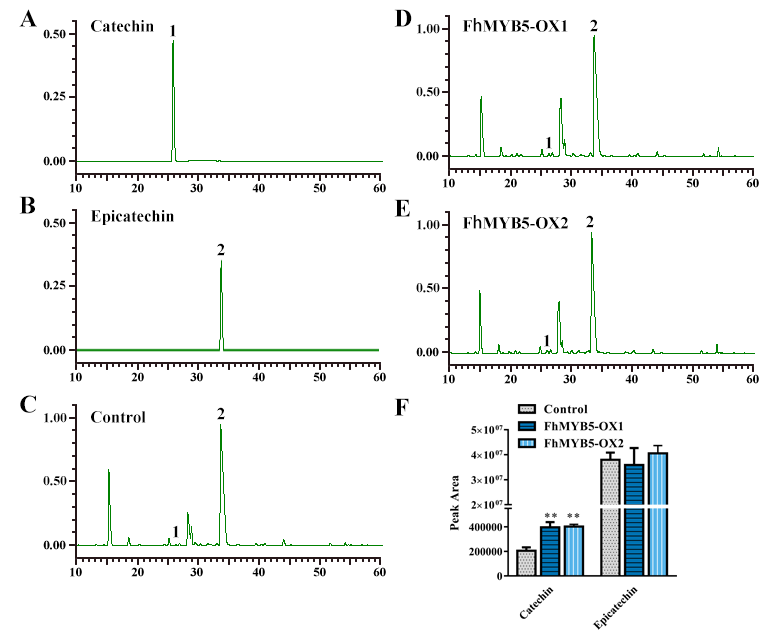


Figure S3. The relative contents of catechins and epicatechins in tobacco stamens detected by HPLC. Control, Wild type stamens; OX, stamens overexpressing *FhMYB5*. The relative contents were quantified by peak area. Data represented means ± SD of three replicates. T-test was carried out to analyze the significant difference (* represented P < 0.05; ** represented P < 0.01).
